# Implementing the Emergency Triage, Assessment and Treatment *plus* admission care (ETAT+) clinical practice guidelines to improve quality of hospital care in Rwandan district hospitals: healthcare workers’ perspectives on relevance and challenges

**DOI:** 10.1186/s12913-017-2193-4

**Published:** 2017-04-07

**Authors:** Celestin Hategeka, Leah Mwai, Lisine Tuyisenge

**Affiliations:** 1 ETAT+ Program, Rwanda Paediatric Association, Kigali, Rwanda; 2 School of Population and Public Health, Faculty of Medicine, University of British Columbia, Vancouver, BC Canada; 3 Maternal and Child Health Program, International Development Research Centre, Ottawa, ON Canada; 4 Afya Research Africa, Nairobi, Kenya; 5 Department of Paediatrics, University Teaching Hospital of Kigali, Kigali, Rwanda

**Keywords:** Implementation, Clinical practical guidelines, ETAT+, Healthcare worker, District hospital, Qualitative research, Rwanda

## Abstract

**Background:**

An emergency triage, assessment and treatment plus admission care (ETAT+) intervention was implemented in Rwandan district hospitals to improve hospital care for severely ill infants and children. Many interventions are rarely implemented with perfect fidelity under real-world conditions. Thus, evaluations of the real-world experiences of implementing ETAT+ are important in terms of identifying potential barriers to successful implementation. This study explored the perspectives of Rwandan healthcare workers (HCWs) on the relevance of ETAT+ and documented potential barriers to its successful implementation.

**Methods:**

HCWs enrolled in the ETAT+ training were asked, immediately after the training, their perspective regarding (i) relevance of the ETAT+ training to Rwandan district hospitals; (ii) if attending the training would bring about change in their work; and (iii) challenges that they encountered during the training, as well as those they anticipated to hamper their ability to translate the knowledge and skills learned in the ETAT+ training into practice in order to improve care for severely ill infants and children in their hospitals. They wrote their perspectives in French, Kinyarwanda, or English and sometimes a mixture of all these languages that are official in the post-genocide Rwanda. Their notes were translated to (if not already in) English and transcribed, and transcripts were analyzed using thematic content analysis.

**Results:**

One hundred seventy-one HCWs were included in our analysis. Nearly all these HCWs stated that the training was highly relevant to the district hospitals and that it aligned with their work expectation. However, some midwives believed that the “neonatal resuscitation and feeding” components of the training were more relevant to them than other components. Many HCWs anticipated to change practice by initiating a triage system in their hospital and by using job aids including guidelines for prescription and feeding. Most of the challenges stemmed from the mode of the ETAT+ training delivery (e.g., language barriers, intense training schedule); while others were more related to uptake of guidelines in the district hospitals (e.g., staff turnover, reluctance to change, limited resources, conflicting protocols).

**Conclusion:**

This study highlights potential challenges to successful implementation of the ETAT+ clinical practice guidelines in order to improve quality of hospital care in Rwandan district hospitals. Understanding these challenges, especially from HCWs perspective, can guide efforts to improve uptake of clinical practice guidelines including ETAT+ in Rwanda.

**Electronic supplementary material:**

The online version of this article (doi:10.1186/s12913-017-2193-4) contains supplementary material, which is available to authorized users.

## Background

Despite international efforts, newborn and child health remains a significant health challenge in low- and middle-income countries (LMICs). Reducing child mortality was one of the eight Millennium Development Goals (MDGs) adopted by the international community (MDG 4: calls for reducing the deaths of children under five years of age by two-thirds between 1990 and 2015). Remarkable progress has been recorded in saving children’s lives globally since 1990- the number of under-five deaths has declined from 12.6 million in 1990 to 6.6 million in 2012, but during the same period neonatal survival has improved more slowly, with 44% of all under-five deaths in 2012 occurring during the neonatal period [1]. Despite progress, neonatal and child mortality remain high in LMICs. In 2013, nearly 6.3 million children under-five died worldwide [2]. Sub-Saharan Africa accounts for nearly half (49%) of the global burden of newborn and child deaths despite being home to just 11% of the global population. Reducing these inequities and saving more children’s lives by ending preventable child deaths should therefore remain a priority on the post-MDGs 2015 agenda. The newly established Sustainable Development Goals (SDGs) seek to build on the MDGs and complete what these did not achieve [3]. In Rwanda, approximately 24,000 children died in 2013 – of whom ~39% were neonates [4]. The leading causes of mortality in children younger than five years in Rwanda are neonatal related complications (e.g., preterm, asphyxia, neonatal sepsis); pneumonia; dehydration/diarrhea and malaria [4–6]. Previous research has also identified gaps in the quality of hospital care provided to sick newborns and children in Rwanda [7].

A major reason for the slow progress in reaching the MDGs in many LMICs is the ‘know-do-gap’– the gap between the existing knowledge on how to reduce the burden of illness and what is implemented [8]. While there is a wealth of evidence on the efficacy and effectiveness of numerous health care interventions in sub-Saharan Africa, there is still relatively limited evidence on how best to implement or scale up such interventions in order to achieve the desired impact [9]. Evidence suggests that a key opportunity for narrowing the know-do gap and accelerating the attainment of the set MDG and SDG targets lies in identifying simple interventions as well as the most optimal ways to train and incentivise an implementation workforce and future scale-up leaders [10].

Advanced pediatric life support trainings have been advocated for implementation in LMICs to contribute to the reduction of under-five mortality in these countries and thus contribute to the achievement of MDG 4 [11]. The ETAT+ training, a locally adapted pediatric life support program, was developed in East Africa for health professionals caring for acutely ill children, and aimed to improve pediatric emergency and admission care in the initial 24–48 h of hospitalization [12]. ETAT+ expands the original World Health Organization (WHO) emergency triage assessment and treatment (ETAT), and is an intensive five-day training covering the recognition and initial management of the commonest medical causes of pediatric hospital admission in East Africa (Table 1). Ayieko and colleagues conducted a cluster-randomized trial in Kenya that provided the state of evidence around the effectiveness of ETAT+ in improving the quality of care for children in low-resource settings [13]. The training was designed to enable healthcare workers to provide important, evidence-based, best-practice care on admission for sick children in resource-limiting settings [12]. Since the introduction of ETAT+ in Rwanda in 2010, in-service healthcare workers and medical students have been trained [14–16].

**Table 1:** Topic covered in the ETAT+ training in Rwanda

Triage
Infant and child resuscitation
Recognition of a sick child
Diarrhea/dehydration and shock
Newborn care – preterm, jaundice, feeding, sepsis
Pneumonia
Malaria
Asthma
Severe malnutrition
Meningitis
Hypoglycaemia
Convulsions
Prescribing and procedures – oxygen, lumbar puncture, intra-osseous
Hospital survey
Morbidity and mortality audit

Clinical practice guidelines (CPGs) such as ETAT+ are developed to help healthcare providers deliver the best care to patients by translating the best available evidence on the management of diseases into specific recommendations for care. Nevertheless, evidence-based guidelines are rarely implemented with perfect fidelity under real-world conditions [7, 14]. Therefore, evaluations of the real-world experiences with implementing such guidelines are important in terms of identifying potential barriers to successful implementation, as well as identifying factors that contribute to their successful adoption and scale-up in various contexts.

Following the demonstration of its effectiveness in Kenya, it was anticipated that the ETAT+ program would be easily adopted to Rwanda given the commonalities in resources as well as epidemiological profiles in the two contexts. However, since the introduction of the program in Rwanda, its relevance and implementation challenges, especially from healthcare workers’ perspective have not been explored. Thus, the current study was undertaken to identify potential challenges to successful implementation of ETAT+ guidelines in Rwandan district hospitals from the healthcare workers’ perspective. Furthermore, the study also explored healthcare workers’ perspective on the relevance of ETAT+ in the Rwanda district healthcare system, including whether they anticipated to change their practice, and which specific aspects of practice they might be willing to change.

### Context

Rwanda – a small, low-income and landlocked country –has a population of 10,515,973 people, of which ~ 85% lives in rural areas [17]. The Rwandan healthcare system is organized along the country’s administrative lay out of 30 districts, with each district having at least one district hospital that operates autonomously and provides healthcare services to well-defined populations in the district [5, 18, 19]. During the 1994 genocide, which claimed the lives of more than 800,000 people including healthcare workers, the Rwandan healthcare system was entirely disrupted [19]. Following this dark period, the government began to rebuild the healthcare system; however, mortality in children younger than five years did not return to pre-1990 rates until 2005 [4]. Actions taken to improve access to primary health services included the restructuring and decentralization of healthcare management in district health facilities as well as developing infrastructure and expanding the community-based health insurance [20]. Currently, access to health services is universal as nearly all Rwandans including the poorest 25% of the population that pay no health insurance premiums, have health insurance [21, 22]. Rwanda has one of the youngest population worldwide, with approximately 48% of its population being younger than 18 years old [17]. While there are approximately 410,100 births per year and only about 20 pediatricians were working in the country as of 2011, mostly in national referral hospitals [5, 16, 23]. Notably, the country has a combined “health – service – provider density” of 8.4 physicians, nurses, and midwives per 10,000 population which falls far below the minimum level recommended by the WHO of 23 providers per 10,000 population [22, 24]. The healthcare workforce in district hospitals is primarily comprised of generalist physicians with six years of basic medical training and nurses with A2-level (secondary school nursing diploma, the lowest level of nursing training available) [22]. These physicians and nurses are often required to handle complicated pediatric and neonatal emergencies in the absence of specialists. Nevertheless, it is noteworthy that efforts are underway to train specialists who will be deployed in all hospitals countrywide [22].

## Methods

The ETAT+ training in Rwanda consists of short lectures on specific topics (Table 1) followed by demonstrations, practical procedures and case based scenarios using mannequins, hospital audit including a review of selected patient medical records within the specific institution where the training is occurring, and assessment and feedback [14]. Discussions and hands on practice take place in small groups of 5–7 participants where all healthcare providers (nurses, midwives and physicians) learn together to promote inter-professional collaboration [14]. The training preparation materials are provided to all participants before the ETAT+ training. These materials include an invitation letter describing the training location and expectation, a training schedule [25], a pre-training knowledge assessment questionnaire, and the ETAT+ clinical practice guidelines disseminated during the training [26]. The ETAT + training for the healthcare workers in Rwanda is run mainly in English language and is completed in five days. Attendance of the training for the entire period is compulsory. Upon completion of the training, all participants retake the knowledge assessment. Further, the participants’ clinical skills are assessed, using an Objective Structured Clinical Examination (OSCE) format, on two clinical skills scenarios (i.e., neonatal resuscitation and management of a severely sick child with shock due to dehydration) [14]. Further details about the ETAT+ training for Rwandan healthcare providers, including effect of the training on knowledge and skills change as well as the associated factors can be found in our previous study (http://journals.plos.org/plosone/article?id=10.1371/journal.pone.0152882#pone.0152882.ref017).

Using a semi-structured questionnaire (Additional file 1), the healthcare workers who were working in Rwandan district hospitals and attended the ETAT+ training between November 2012 and May 2013 were approached and asked, immediately after the training, their perspective regarding (i) relevance of ETAT+ to Rwandan district hospitals (e.g.; “Is ETAT+ relevant to your work?” “What are the most relevant contents?” “Are there other materials that you think should be added to the training?”); (ii) if attending the training would bring about change in their work; and (iii) the challenges they encountered during the training as well as challenges which they anticipated might hamper the translation of the knowledge and skills learnt in the ETAT+ training into everyday practice in order to improve emergency care for severely ill infants and children in their institution. Furthermore, information pertaining to healthcare workers’ characteristics was collected including profession (nurse, midwife, physician); sex; location of district hospital of affiliation (urban or rural); department of affiliation; experience; and whether they had attended any clinical practice guideline disseminating training including ETAT+ before. These healthcare workers wrote down their perspectives / views in French, Kinyarwanda, or English and sometimes mixture of all these languages that are official in the post – genocide Rwanda [27]. Of note, these healthcare workers were informed of the questions throughout the training and encouraged to think about them so that they could provide a more comprehensive list of challenges that needed to be addressed in order to enhance the implementation of ETAT+, in Rwandan district hospitals. Moreover, they were allowed to consult their notes taken over the week of the training.

The healthcare workers’ notes were translated to (if not already in) English and transcribed, and transcripts were imported into NVivo 8 software (QSR International, Doncaster, Australia) for thematic coding and analysis - this approach involves coding data into themes representing the phenomena under investigation [28]. We employed both inductive and deductive approaches to identify themes. Broad themes were developed based on the study objectives/questions that were asked (ETAT+ relevance, anticipated change in their work, challenges encountered during the training, and challenges anticipated to hamper the translation of ETAT+ knowledge and skills into practice). We inductively identified sub-themes within the broad themes. All transcripts were reviewed by two people independently line-by-line identifying anticipated and emerging themes, which were compared and discussed until consensus was reached. The themes that emerged from the analysis are reported with the healthcare workers’ quotes, as appropriate. Further, we reviewed all the ETAT+ training reports, and used the data to complement and corroborate the findings from the analysis of the healthcare workers’ notes, particularly, those relevant to the training delivery challenges. Several themes emerged including: *language barriers, timing/delays in receiving training materials, intense training schedule.*

## Results

### Characteristics of the study sample

Two hundred and eleven healthcare workers were enrolled in the ETAT+ training between November 2012 and May 2013, and were handed a questionnaire including the questions under investigation in the current study. Of these, 171 (81.0%) returned their complete questionnaires that were analyzed in this study. Table 2 shows the characteristics of the healthcare workers included in the current study.

**Table 2:** Characteristics of the study sample

	Overall Study Sample	
	*n* = 171	%
Profession		
Nurse	110	64.4
Midwife	24	14.0
Physician	37	21.6
Language proficiency		
French	92	53.8
English	20	11.7
Bilingual	59	34.5
Sex		
Female	114	66.6
Male	57	33.3
Country region		
Eastern	23	13.4
Kigali City	24	14.0
Northern	47	27.5
Southern	55	32.2
Western	22	12.9
Hospital location		
Rural	64	37.4
Urban	107	62.6
Department of affiliation		
Emergency Room	47	27.5
Maternity	83	48.5
Pediatrics	70	41.0
Other	18	10.5
Experience		
Less than or equal to one year	22	12.9
More than one year	149	87.1
Ever attended CPG^a^ including ETAT+ training before		
Yes	150	87.7
No	21	11.3

^a^CPG, clinical practice guidelines (including ETAT+)

### Relevance of the ETAT+ guidelines to Rwandan district hospitals

Nearly all healthcare workers who were trained in ETAT+ stated that the training was highly relevant to the district hospitals in which they were working, and especially that what they learnt in the training aligned with what they are expected or required to do as part of their work. Many healthcare providers said they wished they had been trained before starting their work appointment in their district hospital.

“This is an important and organized training, relevant to my everyday work.”

“I learned lots of materials relevant to my work in district hospital…. I wish I had participated in this training before my job.”

“The [ETAT+] training uses scenarios similar to what I encounter in my work.”

Nevertheless, some midwives expressed concerns over some materials covered in the training that were not relevant to their work, and thought that the “neonatal resuscitation and feeding” components of the training were more relevant to them than other components of the training. Fourteen of the 18 healthcare workers who were affiliated with any departments other than pediatrics (e.g., emergency room (ER) / outpatient department (OPD), maternity, anesthesia) thought that while the training was interesting, they may not get the opportunity to apply what they had learned until or unless they shifted to relevant departments (e.g., pediatrics/neonatology, ER/OPD).

“I enjoyed the training, however most of the contents covered are not relevant to my work as a midwife”.

“As a midwife, content related to newborn care is most relevant to my work.”

“Although I am currently not based in a unit where I will use what I learnt, I could be shifted anytime to paediatrics”.

While the entire training seemed to be relevant to the district hospitals in Rwanda, some of the contents covered in the training were more highly ranked – these include “triage”, “recognition and management of shock/dehydration”, “management of severe acute malnutrition including feeding”, and “neonatal resuscitation and feeding”. In particular, the mortality and morbidity audit was found to be an important component of the training as it helped healthcare workers to self-audit and learn from errors/mistakes to improve care rendered to severely ill infants and children in their hospitals. However, some healthcare workers stated potential barriers to successful audit including a “culture of blame” predominant in their institution. Overall, the healthcare providers who attended the training showed tremendous enthusiasm to learn in order to better help their patients in their institution.

“I found hospital audit helpful and engaging, as I could see what was done wrong, and discussed with others what could have been done based on [ETAT+ clinical practice guidelines] recommendations.”

“Using the while booklet [Rwanda basic pediatrics protocols] as we were auditing medical record helped integrating [ETAT+] materials.”

“The audit here [in ETAT+ training] seems friendly. We are learning not blaming.”

Ironically, while many of the healthcare workers complained about the intensity of the training (as described below), some of them suggested additional topics in the ETAT+ training. Specifically, these healthcare workers expressed a need to include topics such as “child abuse” (62/171), “pediatric tuberculosis and HIV” (89/171), as well as “interpretation of chest radiography” (42/171). Fifty-two of the 171 also suggested including the ETAT+ training in the formal professional medical and nursing education in Rwanda, or at a minimum, making the ETAT+ training a requirement before working in the pediatrics department (48/171).

### Anticipated change to practice

Within the 171 questionnaires returned, the following areas of anticipated change in practice after attending the ETAT+ training emerged: *(1) Triage, (2) Confidence/reference job aids, (3) resuscitation, (4) prescribing, and (5) feeding*.

- ○ *Triage*

Triage emerged as one of the most favorite topics for all healthcare workers who attended the ETAT+ training. It was consistently ranked as the most important and relevant topic for the district hospitals in Rwanda. Given the shortage of staff and crowded hospitals, the need to identify, based on evidence, those children and infants that need the most attention was recognized. Concurrently, many healthcare workers mentioned that there was no functional triage in their hospitals, and thus were eager to initiate triage system in their institution.

“I now understand what I should be looking for when I have several children queuing at the OPD

“I will always be walking with my white booklet [Rwanda basic pediatrics protocols] to check emergency, priority, and non-emergency/priority signs…even before calling MDs. I will know what to say when I call MDs or transfer [a patient]”.

- ○ *Handing unstable patients*

Most of healthcare workers, particularly nurses who constitute the majority of the workforce in the district hospitals, stated that ‘participating in the ETAT+ training increased their confidence with respect to handling unstable children’. They also expressed how they appreciate the clear and concise instruction and case based scenarios that mimic what they encountered in their practice.

“Now I will be confident when I call for help. I know what to say… I can do preliminary assessment of a severely ill child while waiting for an attending physician to show up”.

“When I started my appointment in the district hospital, I noted that all unstable patients were given oxygen and IV fluids, I did so too…now I believe I will have ideas, for example, how to treat shock, especially how much fluids to give and how to monitor [the patients]”.

“I used to say it’s emergency usually MDs don’t ask why, but in case they ask…I don’t have much to say. Now I know what to document and how to substantiate my decision”.

- ○ *Prescribing and feeding*

In the ETAT+ training, healthcare workers are provided currently recommended protocols including drug prescription guidelines. The training also promotes using reference guidelines, especially when it comes to prescribing to prevent errors. While most healthcare workers acknowledged that they used to prescribe without referring to guidelines, they commended how the ETAT+ training promoted the use of job aids and stated that they would start using these job aids when prescribing any treatment to their patients. Some therapies including oxygen, feeds and gentamicin were consistently listed as not being prescribed based on current recommendations in their hospitals. Most of the trained healthcare workers said gentamicin is prescribed twice or thrice daily while current recommendation is once daily. Some healthcare workers, especially, nurses mentioned that having the white booklet [Rwanda Basic Pediatric Protocols] in their pocket will help not only facilitate discussion with MDs who may be prescribing wrong dosages and or dosing schedules (e.g., gentamicin and oxygen), but also to provide treatment consistent with the Rwandan clinical practice guidelines.

“It’s unbelievable how we have been prescribing gentamicin BID, while current protocol recommend OD as discussed here [in the ETAT+ training]”.

“I had attended another training when we were told how to appropriately prescribe gentamicin, but when discuss with MDs they refuse to abide…now I am happy that I have a reference adopted by our own MOH to use to argue with them. And honestly I think I wont administer it or write in the nurse cardex if I believe it’s a wrong dose”.

“I used to think that one consults protocols because they are mediocre…but now I understand that better quality of care relies on team work, and consulting reference”.

“This booklet [Rwanda basic pediatrics protocols] will be an important guide me to correctly prescribe drugs and feeds to newborns and children with SAM [severe acute malnutrition].”

Moreover, the healthcare workers mentioned inconsistency in prescribing feeding in their health facilities, and some said that there are no clear protocols as things keep on changing depending of the head of departments among other things. Nevertheless, it is noteworthy that these healthcare workers appreciated referring to locally relevant guidelines such as ETAT+ clinical practice guidelines, which they perceived to be more relevant than guidelines from elsewhere which have not been adapted locally. Further, they highlighted that the provision of case based scenarios mimicking what they encountered in their hospitals would facilitate their ability to apply what they had learned in the ETAT+ training.

### Barriers to implementation of the ETAT+ clinical practice guidelines

The analysis of the healthcare providers’ perspectives revealed the following themes and sub-themes related to challenges hindering successful implementation of the CPGs in the district hospitals in Rwanda:

- **Challenges during the ETAT+ training:** (i) Language barriers, (ii) intensity of the training, (iii) onsite training, (iv) shortage of staff & training healthcare workers who are unlikely to use skills/knowledge gained in the ETAT+.
- **Challenges anticipated to hamper successful implementation in the district hospitals**: (i) staff shifting/turnover, (ii) reluctance to change, (iii) limited resources, (iv) knowledge and skills decay, (v) hospital leadership, and (vi) conflicting protocols.

#### Challenges encountered during the ETAT+ training

- ○ *Language barriers*

Language issues were highlighted as one of the major challenges many healthcare workers faced during the ETAT+ training. Because most of the training materials were in English, those who were not fluent in English consistently reported language as a major barrier to keep up with the training.

“Training, and booklet [training materials] are in English that I barely understand”

“I had trouble understanding the pre-training materials because was in English. But at least the training was a mixture of English, French and Kinyarwanda and I could follow”

“The booklet [Rwanda basic pediatric protocols] are interesting but is in English so I write translated term, during the training, that I will be referring to”.

Review of the training reports also revealed language as significant challenges particularly when training healthcare workers who did not identify themselves as fluent in English. While during the training participants could ask questions and require further explanations in Kinyarwanda (mother tongue in Rwanda), there were some participants who did not speak Kinyarwanda, especially some healthcare workers from foreign countries such as DR Congo, who constitute the majority of the foreign medical graduates in Rwanda. Notwithstanding the challenges, some participants shared their enthusiasm stating that the ETAT+ training was one of the best trainings they had ever attended, and indicated that they had felt like they had got an opportunity to return to school and learn, and were eager to go back to their hospitals to help patients.

- ○ *High drop out rates associated with onsite training*

Onsite held ETAT+ trainings were associated with high dropouts, and staffs being required to do some hospital work (e.g., night call) during the intense five-day ETAT+ training. Additionally, for those whose ETAT+ training was held in or close to their facility, hospitals tended to release more staff than they could handle, contributing to dropouts.

“I attended the training held in my hospital, and had been on night call every other day because of limited staff in my department”

“I had to cover night calls in order to attend the training during the day. I was exhausted during the training and could not concentrate enough, and the training itself is so demanding”

- ○ *Challenges associated with the duration and intensity of the training*

A review of the notes of healthcare workers who attended the training as well as the training reports revealed issues related to intensity of the training. The participants suggested revisiting the schedule to extend the training over at least a two-week period. Many participants also suggested minimizing lecturing in the afternoon as they were usually tired and preferred practical sessions. In addition, some healthcare workers stated that they got tired and did not get the chance to revise the material before the following day.

“Too much [material to cover] in just a week”

“There are too much information to learn and a week is really short”.

- ○ *Staff shortage and not sending healthcare workers who would use skills post training.*

While the ETAT+ training is for healthcare providers working in the pediatrics/neonatology, ER, OD, labour and delivery room, some participants from pharmacy, ARV (HIV treatment unit), social workers and other departments also attended the training. These participants mentioned that they were willing to be shifted to departments where they could use the skills/knowledge acquired. Likewise, a review of the training reports revealed similar issues – some participants were less likely to use acquired skills and knowledge in patient care.

“None from ER came because shortage of staff there…so they send me, and I am not likely to use what I learnt here”.

“Sometimes if a workshop is incentivized those in leadership position, decide to attend although they don’t practice”

- ○ *Format and timing of delivery of the training materials.*

Sometimes because of limited budget, training materials were not printed and participants were sent e-copies. Some of these participants mentioned lack of internet and computer to access these materials before the training, and they believed that they could have learned more had they prepared for the training beforehand.

“I don’t have access to internet or computer from home. I can access a computer from the hospital; [however, I am] not there for that purpose”

#### Challenges anticipated to hinder the implementation of ETAT+ in Rwandan district hospitals

Healthcare workers highlighted the following challenges they thought would hamper translating into practice what they learnt to improve patient outcomes: (i) staff shifting/turnover; (ii) reluctance to change; (iii) limited resources; (iv) knowledge and skills decay; (v) hospital leadership; and (vi) conflicting protocols.

- ○ *Staff shifting/turnover*

One of the major issues that healthcare workers highlighted was the high rate of staff shifting/turnover both externally and internally. With respect to within facility (internal) shifting/turnover some healthcare workers noted that although they benefited from trainings they were scheduled to ‘shift’ to new departments where they could not use the skills/knowledge learnt in ETAT+. Likewise, these healthcare workers said they would easily forget what they learnt if they did not get a chance to apply it. In addition, some healthcare workers mentioned their intention to leave their position in the district hospitals for better jobs or studies in the future.

“I was trained in essential neonatal care now I am working in the internal medicine department”.

“[I] trained in ALSO …now working in pharmacy and administration”.

“Although I am attending this training I work in HIV drug unit so I may not use what I am learning here [in the ETAT+ training]”.

- ○ *Conflicting protocols*

The healthcare workers shared their concerns about several conflicting protocols in their hospitals (e.g., neonatal feeding and resuscitation), stating that it was difficult for them to know which one to use. District hospital administration, the MOH and Rwandan healthcare professional associations should ensure consistent and harmonize protocols where necessary.

“There are a lot of guidelines out there, don’t know which one to use or not, particularly in neonatology”

- ○ *Limited resources*

Some of the resources needed to implement ETAT+ were not readily available in their hospitals (e.g., intra-osseous (IO) equipment, metered dose inhaler (MDI) and nebulizers, equipment needed to conduct some investigations) and limited healthcare workforce. For instance, while the healthcare workers found the ETAT+ training component on the “IO access” helpful, most of them mentioned that they had never had a chance of putting an intra-osseous (IO) access/line and were not likely to get a chance to perform the procedure in their institution.

“We practiced how to give an IO infusion, but we don’t have the [IO] equipment in the hospital.”

“I have never seen the IO before this training. I don’t think we have it in my hospital.”

“No nebuliser in my hospital.”

“The [ETAT+] program should provide us some necessary equipment to implement what we learnt.”

*Lack of supervision/mentorship, knowledge/skills decay, and reluctance to change* were also highlighted as important challenges that hindered successful translation of knowledge to practice. Resistance to change was highlighted as a significant challenge to implementing new clinical practice guidelines in Rwandan district hospitals. Healthcare professionals with more experience, especially MDs, along with heads of departments are more reluctant to change if they are not trained or involved in the implementation process.

“Old people, senior staff…particularly MDs if not trained don’t comply with new guidelines compared to nurses”.

“While I learnt a lot of stuff in the training, I will forget them if no follow up/ refresher course”

“We learnt a lot of stuff in trainings but like other trainings I attended it can be difficult to translate what learnt in these trainings to practice without supervision, we would like to have trainers come in our setting to help us”

## Discussion

Proven effective newborn and child health interventions need to be successfully implemented to contribute to sustainable reduction of mortality in children under-five years in low- and middle- income countries. The primary purpose of this study was to identify challenges that healthcare workers anticipated as likely to hinder successful implementation of the ETAT+ clinical practice guidelines in Rwandan district hospitals. In addition, we explored healthcare workers’ perspectives with regards to the relevance to ETAT+ in Rwandan district hospitals, whether these healthcare workers anticipated to change practice, and which specific aspects of practice they might be willing to change following the ETAT+ training. Nearly all participating healthcare workers stated that the training was highly relevant to the district hospitals and that it aligned with their work expectations. However, some midwives believed that the “neonatal resuscitation and feeding” components of the training were more relevant to them than other components. Many healthcare workers anticipated to change practice by initiating a triage system in their hospital and to use job aids including guidelines for prescription and feeding. Most of the anticipated challenges stemmed from perceived ETAT+ dissemination issues (e.g., language barriers, format of training materials, intense training schedule) and health facility related challenges (e.g., staff shifting/turnover, limited resources, reluctance to change, conflicting clinical practice guidelines).

While uptake of clinical practice guidelines is a complex process, key factors including relevance of these guidelines to the system and routine work of healthcare workers can enhance their uptake. For example, Irimu et al.’s ethnographic study conducted in a Kenyan hospital suggested that ETAT+ relevance to routine clinical practice was of the of the factors that facilitated its uptake by healthcare workers in Kenya [29]. Our findings suggest that healthcare workers trained in ETAT+ believe that the training is highly relevant to the Rwandan healthcare system, especially in district hospitals (which constitute the backbone of the Rwandan healthcare system) where healthcare providers who do not have specialty training generally take care of severely sick infants and children without supervision. The ETAT+ training highlights the most common illnesses – leading causes of under-five mortality in Rwanda and in the region – that healthcare workers involved in providing healthcare to sick infants and children would encounter in everyday practice in Rwandan district hospitals [12, 14]. As such, the high relevance of ETAT+, as expressed by Rwandan healthcare workers, could contribute to its successful implementation in Rwandan district hospitals. Rwandan healthcare workers even expressed willingness to make the training a requirement (e.g., recertification) to work in paediatrics and neonatology. Moreover, while recognizing challenges to translate what they learned into practice, trained healthcare workers anticipated to make changes in their practice after the training, including establishing a functional triage system in their facilities and regular use of job aids especially when prescribing drug therapies, which could help bridge gaps in the process of neonatal and pediatric care identified in Rwandan district hospitals [7]. Going forward, complementing the ETAT+ training with regular supervision and mentorship could help not only to ensure that knowledge translation takes place, but also identify further opportunities to enhance the impact of the ETAT+ program.

While it is well known that participating in a training program improves knowledge and skills [14–16, 30], there are barriers that might hamper the use of newly acquired skills, especially in a context where people could be resistant to changing things. For example, a recent study that evaluated the performance of health care providers in the management of seriously sick children in Kenya suggests that educational interventions alone may not be sufficient to deliver high quality care, and effectively adapting interventions to the local context is equally as important [29]. Similarly, Baradaran-Seyed et al.’s study found that one of the major barriers to implementation of clinical practice guidelines in Iran was a healthcare system not designed to easily integrate evidence-based clinical practice guidelines [31]. Arguably, an understanding of local healthcare system organizational factors that can affect healthcare providers’ behavior should guide and inform the implementation of clinical practice guidelines such as ETAT+.

In Rwanda, involving district hospital leadership as well as training large numbers of healthcare workers and use of standards might help bring about change. For example, a district hospital could ensure that all healthcare workers are ‘certified’ in pediatric resuscitation and regular ongoing professional training could be introduced to make sure people retained their skills. An outreach type of program that supports healthcare workers in their own environment and ensures that the necessary equipment is available in good condition, would be timely. This would involve working at the ministry level to develop effective policies and standards for continuing medical education (CME) and health care assessments. Further, engaging all stakeholders involved in clinical practice guidelines development and implementation could help to avoid disseminating conflicting clinical practice guidelines – an important challenge highlighted in our study.

Lack of resources was identified as a significant challenge to successful implementation of the ETAT+ program in our study. This is consistent with previous research in similar settings [11, 32–34]. For example, a survey of Rwandan district hospitals identified limited availability of resources necessary to provide neonatal and pediatric emergency care (e.g., all hospitals surveyed lacked intra-osseous needles for the management of shock and half of the hospitals evaluated lacked BVM for newborns) [34]. In Kenya, English and colleagues found that many essential items for the care of severely ill children were lacking in many district hospitals [33]. Likewise, shortages of drugs, equipment, disposable materials as well as facilities made it difficult to implement sepsis management guidelines in Mongolia [32]. While in the ETAT+ training healthcare workers are taught how to correctly assess children with dehydration/shock and how to resuscitate them with fluid, including putting an intra-osseous (IO) line when necessary, our findings and prior research suggest that some of the required equipment are not available in the hospitals and this may therefore hamper successful implementation of the ETAT+ program in Rwanda [34]. Given that dehydration/shock usually due to diarrheal diseases is one of the leading causes of under-five mortality and morbidity in Rwanda [4–6] and the evidence from prior research recommending IO access if IV cannot be promptly established, and suggesting that IO access may be ‘easily established’ by healthcare workers with little training and is ‘more rapidly achieved’ than IV access, IO access equipment should be made readily available in the district hospitals [35, 36].

The ETAT+ clinical practice guideline dissemination related challenges (e.g., format of course materials, location of the training) and healthcare workers’ language proficiency have been suggested as correlates of healthcare providers’ performance in the ETAT+ training in Rwanda [14]. Rwanda shifted its official language from French to English in 2008 [37], and secondary and post-secondary education and CME programs are run primarily in English. The current study findings, using qualitative methods, are in line with findings from our previous quantitative study that used within the ETAT+ training metrics to explore potential factors associated with performance of Rwandan healthcare providers in ETAT+ [14]. It was found that relative to healthcare workers who identified as proficient in French, those who identified as proficient in both English and French had on average a higher improvement in knowledge and were more than twice likely to pass the practical skills assessment. This discrepancy might be explained by challenges expressed by healthcare providers who were not proficient in English (e.g., unable to adequately prepare or understand course content, if it was taught mainly in English). In addition, low computer ownership and internet penetration in rural areas [38] might explain challenges experienced by healthcare workers from rural areas in the preparation for the training when they were not provided printed training materials. While ETAT+ training held within health facilities was cost saving (e.g., costs associated with accommodation of participants and training venue were saved), it was found as in a previous study, to be associated with a poorer performance, which may be due to the fact that healthcare workers may have been required to continue to be involved in some of the work-related activities (e.g., direct patient care, night call) during training time and could have missed important material when away [14, 15]. Going forward, we believe that printed training materials should be provided to ETAT+ training participants and these materials should be available in a language that participants understand. Moreover, efforts should be made to organize trainings in French or English *separately* to accommodate participants’ language proficiencies. Further, for each training, participants could be recruited from across many district hospitals so as not to put excessive personnel absence or strain on any single hospital to require those attending intensive training such as ETAT+ to cover night calls. Equally important, ETAT+ organizers should communicate to the hospitals early about the training so that staff rotation can be modified to accommodate absences for training.

Prior research suggested a number of factors (e.g., staff turner-over, knowledge/skills decay) to be significant barriers to the translation of knowledge and skills to practice [16, 39–42]. For example, a recent study by Tuyisenge found that 62.5% of healthcare workers from the district hospitals in the Eastern Province of Rwanda that were trained in Advanced Life Support in Obstetrics (ALSO®) between October 2012 and October 2013 had left their work in the district hospitals by August 2014 for various reasons including taking better job/position (26.6%) and furthering their studies (42.2%) [39]. Clearly, it is possible that some of the healthcare workers trained in ETAT+ may stop working in the district hospitals for various reasons as well. Thus, given the need for a better trained health workforce along with significant resources (both international and local) invested in training healthcare providers, there is an urgent need to evaluate strategies to retain healthcare providers in district hospitals in Rwanda, especially in remote areas. Moreover, efforts should be deployed to prevent internal staff shifting (i.e. staff shifting from one department to another) as this would potentially affect the fidelity of the ETAT+ implementation in Rwandan district hospitals as trained healthcare workers may not be working in departments where their skills and knowledge are most valuable.

Further, it is critical that participants who attend the ETAT+ training are selected from the healthcare workers working in the departments (e.g., paediatrics, neonatology, delivery room, emergency room) where ETAT+ knowledge and skills gained could be put into practice to benefit patients. Clearly, training healthcare workers who do not work in relevant departments may hamper the successful implementation of ETAT+ even if physical resources (e.g., IO) were available in the district hospitals, as these resources would not be appropriately used to benefit patients. As such, ETAT+ training organizers should work with district hospital administration to establish a system to ensure that healthcare workers attending the training work in relevant departments.

## Conclusions

The study findings provide evidence on factors that can hamper successful implementation and scale up of the ETAT+ clinical practice guidelines in Rwandan district hospitals. These factors need to be taken in account when implementing ETAT+ and other continuing medical education interventions within the Rwandan context. However, our findings should be interpreted in light of a number of limitations. The researcher's presence during data gathering, may have affected the participants’ responses. Moreover, typing and translating healthcare workers’ notes may have introduced bias. Further, the current study findings are drawn from data collected about three years ago and, therefore, it is possible that some of our findings may not reflect exactly the current reality on ground. For example, the ETAT+ training materials have been translated to French to accommodate participants’ language proficiencies, and therefore language may not currently be a significant barrier to ETAT+ guidelines dissemination. Despite the age of the data, our findings from a representative sample of healthcare providers working in Rwandan district hospitals, are still relevant – especially as the ETAT+ program is currently been scaled up nationally – and may be generalized countrywide and to other settings with similar context. In particular, the findings of our study suggest that the durability of continued ETAT+ training efforts will be dependent on ensuring that training content is better targeted to selected participants, and in ensuring that those who receive the training are facilitated to apply the knowledge and skills they acquire.

## Additional file

Additional file 1: ETAT+ evaluation questionnaire completed by participants immediately following the ETAT+ training. (DOCX 68 kb)
