# Supplementary material for: Implementing the Emergency Triage, Assessment and Treatment plus admission care (ETAT+) clinical practice guidelines to improve quality of hospital care in Rwandan district hospitals: healthcare workers’ perspectives on relevance and challenges
Source: BMC Health Serv Res. 2017 Apr 7;17:256. doi: 10.1186/s12913-017-2193-4 (PMC5385061; doi:10.1186/s12913-017-2193-4)
Supplement: Additional file 1: — ETAT+ evaluation questionnaire completed by participants immediately following the ETAT+ training. (DOCX 68 kb) [file 12913_2017_2193_MOESM1_ESM.docx]

Kindly provide feedback on the ETAT+ training using the following headings:

1. Training preparation
2. Did you get the reading materials before the training started?
3. What do you think can be done to improve preparation of participants for the training
4. Training delivery

I was satisfied with the following session:

**Lectures**: 1. Strongly disagree 2. Disagree 3. Neither agree nor disagree 4. Agree 5. Strongly agree

**Skills**: 1. Strongly disagree 2. Disagree 3. Neither agree nor disagree 4. Agree 5. Strongly agree

**Scenarios**: 1. Strongly disagree 2. Disagree 3. Neither agree nor disagree 4. Agree 5. Strongly agree

**Hospital surveys**: 1. Strongly disagree 2. Disagree 3. Neither agree nor disagree 4. Agree 5. Strongly agree

**Hospital reports/discussions**: 1. Strongly disagree 2. Disagree 3. Neither agree nor disagree 4. Agree 5. Strongly agree

1. What were the most relevant topics to you?
2. What were the most relevant topics to your hospital and your Healthcare system? (Please include change you anticipate if any)
3. Which topics would you like added to the training?
4. Which topics would you suggest be dropped from this training?
5. How did you feel about the English and French presentation projections?

8. Please list suggestions for improvement of this training

9. Which aspects of the training did you appreciate most?

10. Which aspects of the training did you appreciate least?

11. ANY OTHER COMMENTS? (Please include challenges to successful implementation of ETAT+)
